# Defining the proteome of bone marrow plasma in multiple myeloma and monoclonal gammopathy of undetermined significance

**DOI:** 10.1038/s41408-025-01417-3

**Published:** 2025-11-21

**Authors:** Joel-Sean Hsu, Udit Yadav, Kishore Garapati, Kiran K. Mangalaparthi, Yogesh Chawla, J. Erin Wiedmeier-Nutor, Rafael Fonseca, P. Leif Bergsagel, S. Vincent Rajkumar, Shaji Kumar, Wilson I. Gonsalves, Akhilesh Pandey, Richard K. Kandasamy

**Affiliations:** 1 Department of Biochemistry and Molecular Biology, Mayo Clinic, Rochester, MN USA; 2 Division of Hematology and Oncology, Mayo Clinic Arizona, Phoenix, AZ USA; 3 Department of Laboratory Medicine and Pathology, Mayo Clinic, Rochester, MN USA; 4 Manipal Academy of Higher Education, Manipal, Karnataka India; 5 Division of Hematology, Mayo Clinic, Rochester, MN USA; 6 Center for Individualized Medicine, Mayo Clinic, Rochester, MN USA; 7 Department of Immunology, Mayo Clinic, Rochester, MN USA; 8 Department of Quantitative Health Sciences, Mayo Clinic, Rochester, MN USA

**Keywords:** Myeloma, Cancer microenvironment

**Dear Editor**,

Plasma cell neoplasms encompass a range of disorders, including multiple myeloma (MM) and its precursor condition, monoclonal gammopathy of undetermined significance (MGUS). While prior studies have explored the proteomes of malignant plasma cells, a comprehensive analysis of the proteome of the bone marrow (BM) microenvironment as reflected in BM plasma remains limited [1].

Proteomic profiling of BM plasma presents a promising approach for studying the tumor microenvironment in plasma cell disorders. However, mass spectrometry-based plasma proteomics is hindered by the wide dynamic protein range—high-abundance proteins such as albumin and immunoglobulins can obscure low-abundance proteins. To overcome these challenges, we employed the Olink platform, which offers a broad dynamic range of detection for identifying novel biomarkers and potential therapeutic targets in MGUS and MM [2].

We analyzed the proteome of BM aspirates collected from 10 MGUS patients, 8 MM patients, and 5 healthy controls (Fig. 1A; patient characteristics are described in Supplementary Table S1). The proteins were analyzed based on differential abundance between these groups. Binary comparisons identified 568, 700, and 285 differentially abundant proteins (*p* value < 0.05) between control and MGUS, control and MM, as well as MGUS and MM (Supplementary Fig. S1A–C), respectively. The top 50 differentially abundant proteins ranked by one-way ANOVA were selected for hierarchical clustering analysis (Fig. 1B). Interestingly, unsupervised hierarchical clustering showed a clear separation between the control and the disease groups. In addition, all samples, except for one (MM8), are clustered within the same disease sub-group. This indicates that the top differentially expressed proteins could serve as potential markers for evaluating the progression of plasma cell neoplasms.

**Fig. 1:**
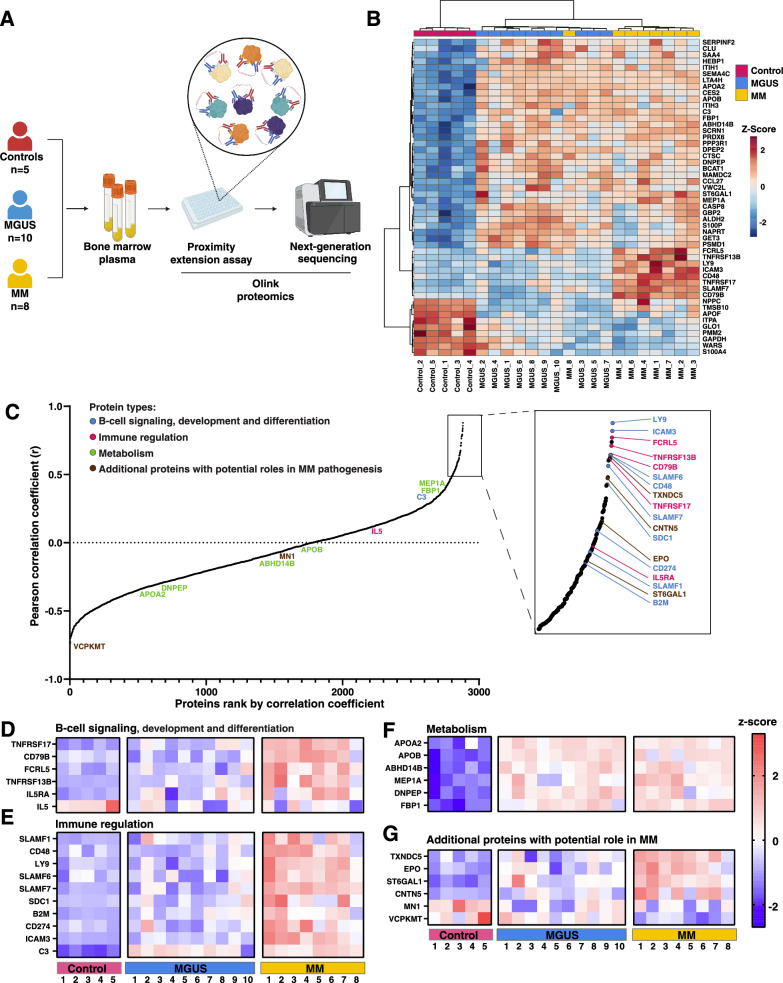
Summary of the proteomic analysis of BM plasma. **A** Schematic overview of the workflow for Olink analysis of BM plasma. Samples from control, MGUS, and MM patients were analyzed using Olink proximity extension assays, and protein abundance was quantified by next-generation sequencing **B** Heatmap displaying unsupervised hierarchical clustering of the top 50 significantly altered proteins ranked by one-way ANOVA. Samples are annotated by group; protein abundance levels are color-coded by z-score, and gene symbols are indicated. **C** Waterfall plot displaying Pearson correlation coefficients between protein abundance and plasma cell burden in bone marrow samples. Known and potential biomarkers are highlighted in distinct colors according to their functional roles. Heatmaps displaying protein abundance of **D** B-cell signaling proteins, **E** Immune-regulating proteins, **F** Metabolic proteins, **G** Additional proteins with potential role in MM. Sample groups are annotated; protein abundance levels are color-coded by z-score, and gene symbols are indicated.

To assess whether plasma cell burden affects protein abundance in BM plasma, correlation analysis of protein abundance and bone marrow plasma cell percentage was performed for MGUS and MM samples (Fig. 1C and Supplementary Table S2). Notably, several B-cell signaling and immune regulatory proteins showed strong correlation with plasma cell percentage, suggesting the plasma cell burden could affect the abundance of these proteins in the BM microenvironment.

BM plasma proteomics also revealed an elevated abundance of B-cell signaling proteins, including TNFRSF17 (BCMA), FCRL5, TNFRSF13B (TACI), and CD79B in MM than MGUS and controls (Fig. 1D and Supplementary Fig. S2). They also correlate strongly with BM plasma cell percentage (Fig. 1C). Notably, BCMA is a current therapeutic target in MM, while anti-FCRL5 agents are under development [3]. CD79B remains unexplored in MM but is studied in other B-cell malignancies [4]. Interestingly, IL5 level decreased, while IL5RA increased with MM progression. IL5’s role in MM remains unclear. However, IL5RA has been reported to be upregulated in MM compared to smoldering MM [5].

Immune regulators, including SLAMF1, CD48 (SLAMF2), LY9 (SLAMF3 or CD229), SLAMF6, SLAMF7 (CS1), SDC1 (CD138), ICAM3, B2M, CD274 (PD-L1) and C3, are highly expressed in MM (Fig. 1E and Supplementary Fig. S3). The expression levels of CD48, LY9, SLAMF6, SLAMF7, SDC1 and ICAM3 strongly correlate with BM plasma cell percentage (Fig. 1C). Notably, SLAMF7, SDC1 and B2M are known plasma cell markers [3]. And CD48 and LY9 are currently being investigated as targets for anti-MM therapy [6, 7]. An increased level of CD274 and ICAM3 suggests potential immune evasion of MM. Furthermore, we found complement C3 significantly more abundant in both MGUS and MM patients compared to controls, indicating that C3 upregulation occurs in the early stages of the disease.

Several proteins involved in metabolic pathways, including lipid and cholesterol transporters (APOA2, APOB), protein-processing enzymes (ABHD14B, MEP1A, DNPEP), and a glucose metabolism enzyme (FBP1), were significantly elevated in MGUS and MM (Fig. 1F and Supplementary Fig. S4). While lipoprotein levels have been studied in peripheral blood in myeloma [8, 9], their link to MM progression is unclear. The metabolic proteins showed no significant difference between MGUS and MM and did not correlate with tumor burden (Fig. 1C). These findings suggest that metabolic alterations occur early in the pathogenesis of plasma cell neoplasms and could serve as early-stage biomarkers.

We also observed several previously unreported proteins with potential roles in the pathogenesis of MM that showed significant changes in protein level in MM development. These proteins include TXNDC5, EPO, ST6GAL1, CNTN5, MAP2, SEMA4C, EDA2R, MN1 and VCPKMT (Fig. 1C and Supplementary Fig. S5). TXNDC5 and EPO have been reported to be upregulated in hypoxic and anemic conditions as well as in MM [10, 11]. Our results also show a higher abundance of TXNDC5 and EPO in MM, with the protein abundance correlating to the tumor burden. We also observed an increased expression of ST6GAL1 and CNTN5 in MM with a moderate to strong correlation to the tumor burden (Fig. 1C). Both ST6GAL1 and CNTN5 have been reported to be upregulated in B-cell malignancies, including MM [12, 13]. Notably, MN1 and VCPKMT are downregulated during disease progression, with VCPKMT showing a strong negative correlation to plasma cell percentage in the BM (Fig. 1C). However, the role of MN1 and VCPKMT in MM is yet to be explored.

To investigate the cellular origins of potential MM and MGUS biomarkers, we analyzed single-cell RNA expression data from Human Protein Atlas, comprising 31 single-cell RNA-seq datasets, focusing on secreted and membrane proteins identified as potential biomarkers in our bone marrow plasma proteome analysis. Specifically, we examined the RNA expression of these candidate markers across common bone marrow cell types, including T cells, B cells, plasma cells, NK cells, dendritic cells, granulocytes, macrophages, monocytes, erythroid cells, fibroblasts, and adipocytes (Fig. 2A, B). As expected, the expression of known plasma cell markers such as TNFRSF17, FCRL5, SLAMF7, and SDC1 shows high plasma cell specificity. Notably, TNFRSF13B, SLAMF1, and ST6SAL1 are also highly expressed in plasma cells, suggesting that they could be further explored as potential therapeutic targets and plasma cell markers.

**Fig. 2:**
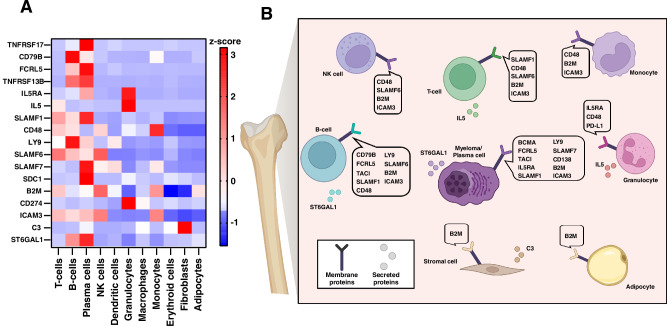
Cellular expression of potential cell surface and secreted biomarkers in the BM microenvironment. **A** Heatmap showing RNA expression of selected biomarkers across cell types in the BM microenvironment. Expression levels are color-coded by z-score; gene symbols are indicated. **B** Schematic illustration of the cellular localization of selected biomarkers within the BM microenvironment.

In this study, we demonstrated that BM plasma can be successfully used to identify protein markers for distinguishing different stages of plasma cell neoplasms. We could detect the higher abundance of currently utilized clinical MM biomarkers and therapeutic targets such as BCMA, SDC1, SLAMF7, and B2M. We also identified potential novel therapeutic targets, such as CD79B and LY9. Anti-CD79B agents are now being developed for B-cell lymphomas and could be repurposed for MM treatment [4]. LY9 shows preclinical CAR-T efficacy in MM cell lines and mouse models [7]. Moreover, we identified proteins, including C3 and several metabolic proteins, that could serve as biomarkers for the detection of early plasma cell neoplasms.

Interestingly, while a previous study reported differences in metabolites such as branched-chain amino acids, tryptophan catabolites, and lipid composition between MGUS and MM bone marrow microenvironments, we observed that many metabolic proteins were present at similar levels in MGUS and MM BM plasma [14]. One explanation could be that the expression of metabolic enzymes may not be directly associated with metabolite levels. Another explanation could be that there are intact compensation mechanisms of metabolic pathways in MGUS clonal plasma cells, which are subsequently lost in more malignant conditions, with more accumulated gene alterations, such as MM, leading to differential metabolite levels in the BM plasma.

A recent Olink proteomics study utilizing samples from UK Biobank investigated protein abundance in peripheral blood plasma and identified ten potential predictors of MM: SLAMF7, TNFRSF17, QPCT, LY9, SLAMF1, CNTN5, TNFRSF13B, TNFSF13, TNFSF13B, and TIMP1 [15]. Notably, many of these proteins, including SLAMF7, TNFRSF17, QPCT, LY9, SLAMF1, CNTN5, and TNFRSF13B, were also found in our study to be more abundant in the bone marrow plasma of MM patients, suggesting that their elevated levels are not confined to the bone marrow microenvironment. However, we did not observe significant differences in the levels of TNFSF13, TNFSF13B, and TIMP1 between MM and control bone marrow plasma samples. This discrepancy may reflect distinct regulatory mechanisms or protein dynamics between the bone marrow and peripheral blood environments.

This is the first study to utilize the Olink platform to evaluate the proteome of the BM microenvironment in a spectrum of plasma cell disorders. While the findings in this study are noteworthy, there are limitations stemming from the small sample size and the heterogeneous clinical nature of MM. Additionally, age differences between control and disease groups could influence certain protein expression levels, potentially confounding some observations. Further validation of these novel biomarkers in the BM microenvironment with a larger patient cohort could open potential applications that include detecting disease, especially in conditions such as non-secretory MM, where there is no circulating paraprotein. Future prospective studies or retrospective studies on paired stored samples to monitor the dynamic changes of these novel biomarker levels from the same individuals as they progress from MGUS to MM in the future would further support their role in monitoring the pathogenesis of MM.

## Supplementary information

Supplementary Table S1

Supplementary Table S2

Supplementary Information

Supplementary Fig. S1

Supplementary Fig. S2

Supplementary Fig. S3

Supplementary Fig. S4

Supplementary Fig. S5

## Data Availability

The data are available upon request from the corresponding authors.
