# Supplementary material for: Defining the proteome of bone marrow plasma in multiple myeloma and monoclonal gammopathy of undetermined significance
Source: Blood Cancer J. 2025 Nov 21;15(1):202. doi: 10.1038/s41408-025-01417-3 (PMC12639027; doi:10.1038/s41408-025-01417-3)
Supplement: Supplementary file 1 — Supplementary Table S1 [file 41408_2025_1417_MOESM1_ESM.docx]

**Supplementary Table 1. Clinical and laboratory characteristics of patients and control samples**

| **Sample** | **Age** | **BMI** | **BM plasma cell %** | **Structural variant** | **Risk factor** | **Gender** | **Race/ethnicity** |
| --- | --- | --- | --- | --- | --- | --- | --- |
| Control_1 | 24 | 24 | N/A | N/A | N/A | Female | Caucasian |
| Control_2 | 28 | 22.7 | N/A | N/A | N/A | Female | Non-Caucasian |
| Control_3 | 43 | 27.6 | N/A | N/A | N/A | Female | Caucasian |
| Control_4 | 33 | 19.6 | N/A | N/A | N/A | Female | Caucasian |
| Control_5 | 24 | 24.7 | N/A | N/A | N/A | Male | Caucasian |
| MGUS_1 | 44 | 23.7 | 5 | N/A | N/A | Male | Caucasian |
| MGUS_2 | 71 | 25.6 | 9 | N/A | N/A | Male | Caucasian |
| MGUS_3 | 68 | 20.8 | 5 | N/A | N/A | Male | Caucasian |
| MGUS_4 | 71 | 35.6 | 5 | N/A | N/A | Male | Caucasian |
| MGUS_5 | 74 | 29.4 | 7 | N/A | N/A | Male | Caucasian |
| MGUS_6 | 45 | 23.1 | 5 | N/A | N/A | Male | Caucasian |
| MGUS_7 | 83 | 26.5 | 5 | N/A | N/A | Female | Caucasian |
| MGUS_8 | 71 | 20.5 | 9 | N/A | N/A | Female | Caucasian |
| MGUS_9 | 82 | 21.5 | 5 | N/A | N/A | Female | Caucasian |
| MGUS_10 | 50 | 25.7 | 5 | N/A | N/A | Male | Caucasian |
| MM_1 | 76 | 27.6 | 70 | Trisomies, del17p | HR | Female | Caucasian |
| MM_2 | 82 | 33.6 | 60 | t(14;16), +1q | HR | Male | Caucasian |
| MM_3 | 72 | 23.9 | 30 | +1q | HR | Male | Caucasian |
| MM_4 | 66 | 26.8 | 30 | Trisomies | SR | Male | Caucasian |
| MM_5 | 66 | 42.9 | 50 | t(11;14) | SR | Female | Caucasian |
| MM_6 | 73 | 20.9 | 50 | +1q, del 17p, trisomies | HR | Female | Caucasian |
| MM_7 | 65 | 30.3 | 66 | +1q, trisomies | HR | Female | Caucasian |
| MM_8 | 29 | 27.5 | 20 | +Myc rearrangement | SR | Male | Caucasian |

BM, bone marrow; N/A, not applicable; HR, High risk; SR, Standard risk.
