# Supplementary material for: Defining the proteome of bone marrow plasma in multiple myeloma and monoclonal gammopathy of undetermined significance
Source: Blood Cancer J. 2025 Nov 21;15(1):202. doi: 10.1038/s41408-025-01417-3 (PMC12639027; doi:10.1038/s41408-025-01417-3)
Supplement: Supplementary file 2 — Supplementary Table S2 [file 41408_2025_1417_MOESM2_ESM.docx]

**Supplementary Table 2. Pearson correlation between selected protein abundance and BM plasma cell percentage**

| **Protein** | **Pearson *r*** |
| --- | --- |
| TNFRSF17 | 0.780226 |
| CD79B | 0.792921 |
| FCRL5 | 0.839264 |
| TNFRSF13B | 0.817692 |
| IL5RA | 0.541293 |
| IL5 | 0.07498 |
| SLAMF1 | 0.523699 |
| CD48 | 0.785778 |
| LY9 | 0.878403 |
| SLAMF6 | 0.792629 |
| SLAMF7 | 0.763333 |
| SDC1 | 0.713781 |
| B2M | 0.506342 |
| CD274 | 0.589591 |
| ICAM3 | 0.857588 |
| C3 | 0.31039 |
| APOA2 | -0.37389 |
| APOB | -0.0697 |
| ABHD14B | -0.12732 |
| MEP1A | 0.420864 |
| DNPEP | -0.34548 |
| FBP1 | 0.386006 |
| TXNDC5 | 0.782216 |
| EPO | 0.608027 |
| ST6GAL1 | 0.520291 |
| CNTN5 | 0.729907 |
| MN1 | -0.11332 |
| VCPKMT | -0.7609 |
