# Supplementary material for: Defining the proteome of bone marrow plasma in multiple myeloma and monoclonal gammopathy of undetermined significance
Source: Blood Cancer J. 2025 Nov 21;15(1):202. doi: 10.1038/s41408-025-01417-3 (PMC12639027; doi:10.1038/s41408-025-01417-3)
Supplement: Supplementary file 3 — Supplementary Information [file 41408_2025_1417_MOESM3_ESM.docx]

**Supplementary Information**

**Materials and Methods**

**Sample processing**

We prospectively evaluated patients with MM and MGUS who underwent BM aspirations of their posterior iliac crest as part of their standard of care clinical evaluation and who provided informed consent, allowing the use of their BM aspirate samples for this research project. The International Myeloma Working Group (IMWG) diagnostic criteria were applied to confirm the diagnosis of MGUS and MM. Their BM plasma was extracted from the BM aspirate by centrifuging the aspirate sample at 2,400 g for 10 minutes before isolating the supernatant into a separate tube. The supernatant was then centrifuged at 2,400 g for 10 minutes before isolating the plasma and storing it in EDTA tubes at −80 °C. The control samples were collected from posterior iliac crest biopsies of individuals without plasma cell dyscrasias and were processed and stored using the same workflow as MM and MGUS samples to isolate the plasma.

**Olink analysis**

The Olink Explore 3072 panel assay (Olink Proteomics AB, Uppsala, Sweden), which utilizes proximity extension assay, was employed for BM plasma proteome analysis. The protein intensity readout is measured by next-generation sequencing on a NovaSeq 6000 sequencer (Illumina). The raw output data is quality controlled, normalized and converted into Normalized Protein eXpression (NPX) values on a log2-scale.

**Bioinformatics and statistical analysis**

Relative protein abundance (NPX) values were transformed to linear values for protein differential analysis. The *p*-values were obtained using unpaired two-tailed t-tests. NPX values were used for unsupervised hierarchical clustering analysis by MetaboAnalyst 6.0(1, 2). GraphPad Prism (10.4.1) was used for graphic construction and statistical analysis. Single-cell RNA-Seq datasets were obtained from Human Protein Atlas (proteinatlas.org)(3)

**References**

1. Ewald JD, Zhou G, Lu Y, Kolic J, Ellis C, Johnson JD, et al. Web-based multi-omics integration using the Analyst software suite. Nature Protocols. 2024;19(5):1467-97.

2. Pang Z, Lu Y, Zhou G, Hui F, Xu L, Viau C, et al. MetaboAnalyst 6.0: towards a unified platform for metabolomics data processing, analysis and interpretation. Nucleic Acids Research. 2024;52(W1):W398-W406.

3. Karlsson M, Zhang C, Méar L, Zhong W, Digre A, Katona B, et al. A single-cell type transcriptomics map of human tissues. Sci Adv. 2021;7(31).

**Supplementary Figure Legends**

**Supplementary Fig. S1. Differential protein abundance of BM plasma between plasma cell neoplasm stages.** Volcano plots showing differential protein abundance in BM plasma between (A) MGUS vs. control, (B) MM vs. control, and (C) MM vs. MGUS. Horizontal dashed lines indicate the significance threshold (*p* < 0.05).

**Supplementary Fig. S2. Proteomic profile of B-cell associated proteins.** Box plots showing the relative abundance of proteins involved in B-cell signaling, development and differentiation. Each dot represents an individual sample. Whiskers indicate the lowest and highest values within each group. *p*-values calculated using one-way ANOVA are shown above the bars.

**Supplementary Fig. S3. Proteomic profile of immune regulation-related proteins.** Box plots showing the relative abundance of proteins involved in immune regulation. Each dot represents an individual sample. Whiskers indicate the lowest and highest values within each group. *p*-values calculated using one-way ANOVA are shown above the bars.

**Supplementary Fig. S4. Proteomic profile of metabolism-associated proteins.** Box plots showing the relative abundance of proteins involved in the metabolic process. Each dot represents an individual sample. Whiskers indicate the lowest and highest values within each group. *p*-values calculated using one-way ANOVA are shown above the bars.

**Supplementary Fig. S5. Proteomic profile of other novel proteins.** Box plots showing the relative abundance of proteins with potential roles in MM progression. Each dot represents an individual sample. Whiskers indicate the lowest and highest values within each group. *p*-values calculated using one-way ANOVA are shown above the bars.
