# Supplementary figures and images for: Defining the proteome of bone marrow plasma in multiple myeloma and monoclonal gammopathy of undetermined significance
Source: Blood Cancer J. 2025 Nov 21;15(1):202. doi: 10.1038/s41408-025-01417-3 (PMC12639027; doi:10.1038/s41408-025-01417-3)

Supplementary Figure 1

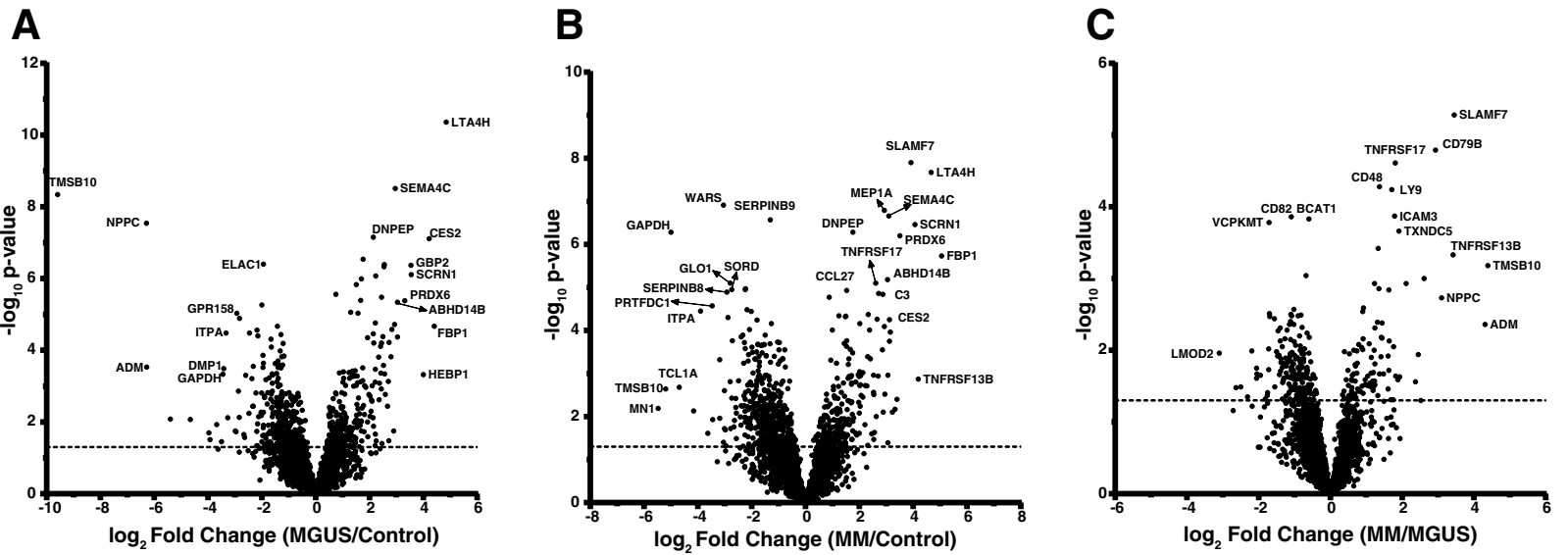

Supplement: Supplementary file 4 — Supplementary Fig. S1 [file 41408_2025_1417_MOESM4_ESM.pdf]

# Supplementary Figure 2

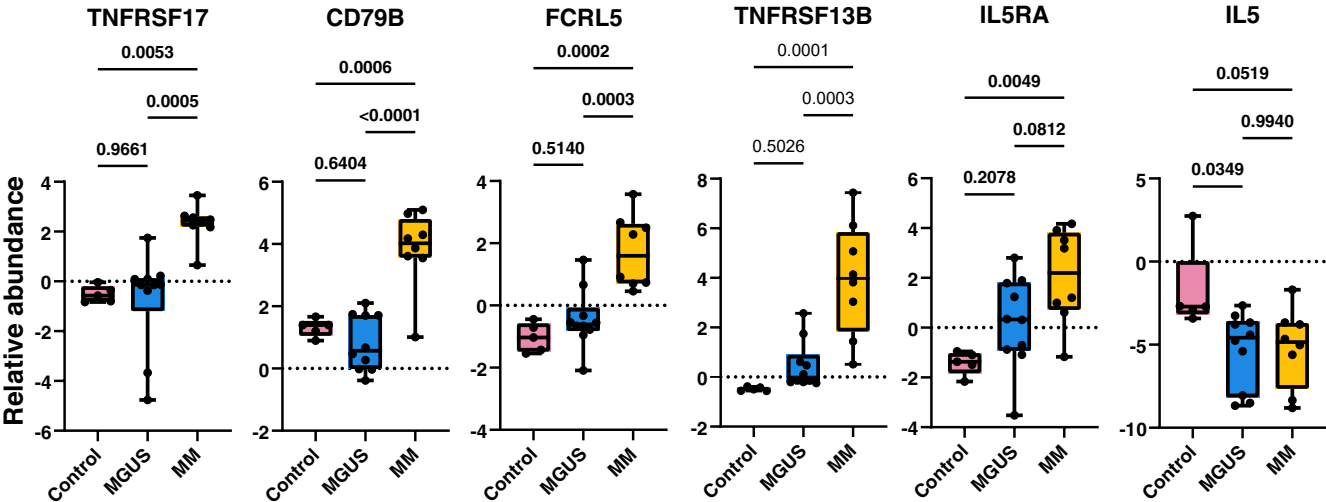

Supplement: Supplementary file 5 — Supplementary Fig. S2 [file 41408_2025_1417_MOESM5_ESM.pdf]

# Supplementary Figure 3

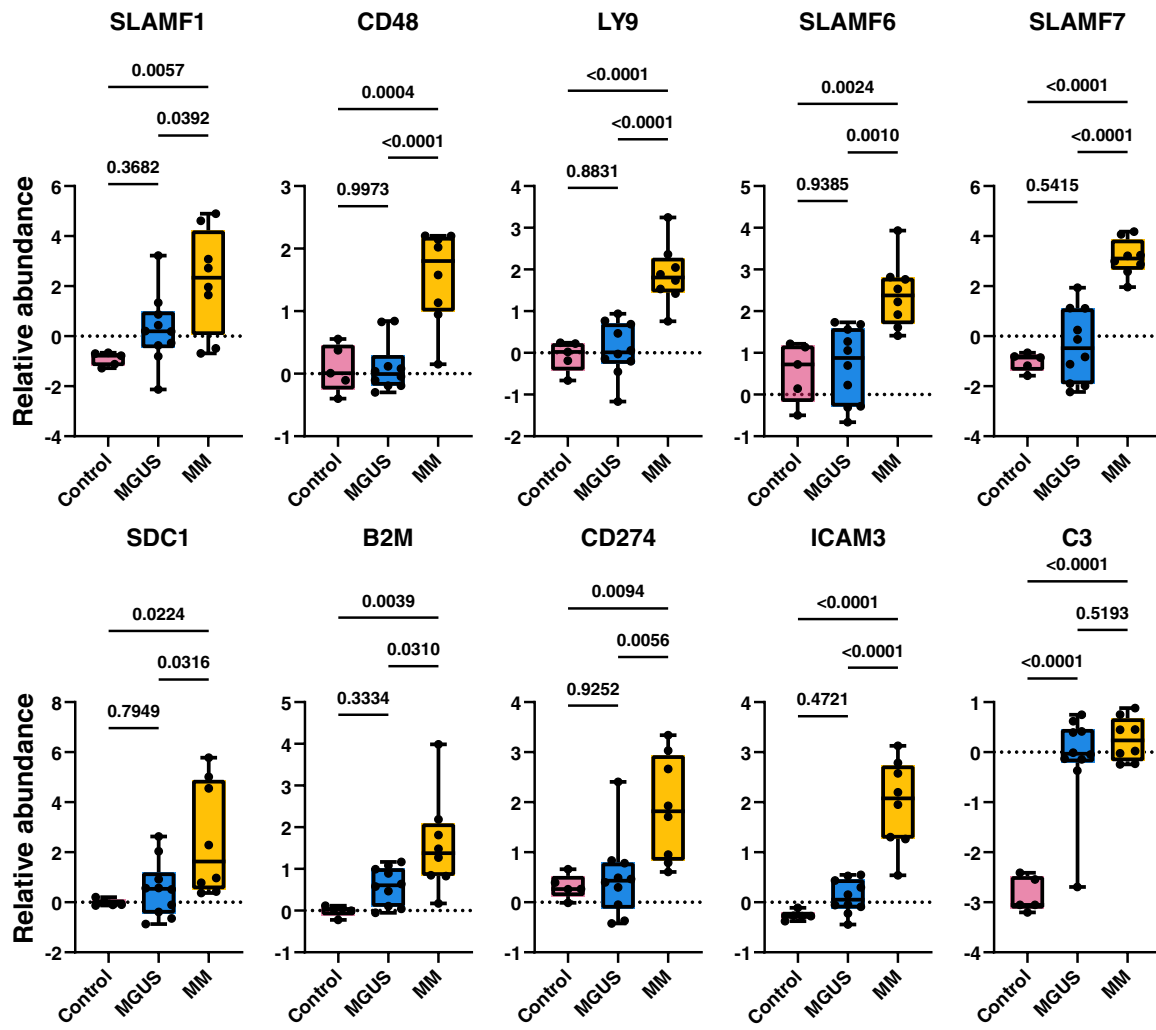

Supplement: Supplementary file 6 — Supplementary Fig. S3 [file 41408_2025_1417_MOESM6_ESM.pdf]

# Supplementary Figure 4

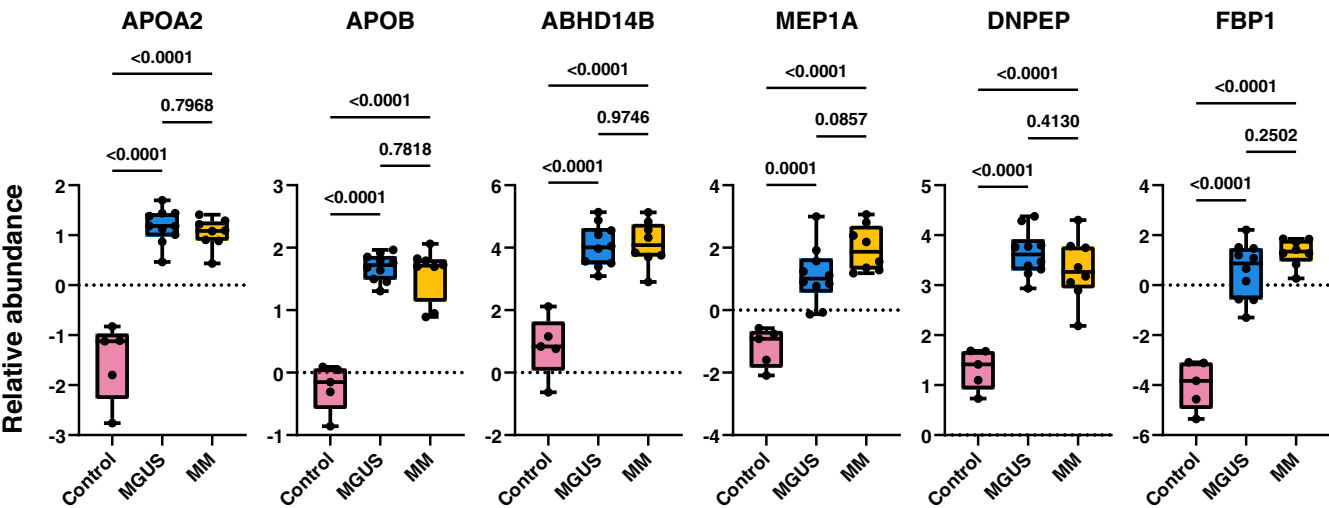

Supplement: Supplementary file 7 — Supplementary Fig. S4 [file 41408_2025_1417_MOESM7_ESM.pdf]

# Supplementary Figure 5

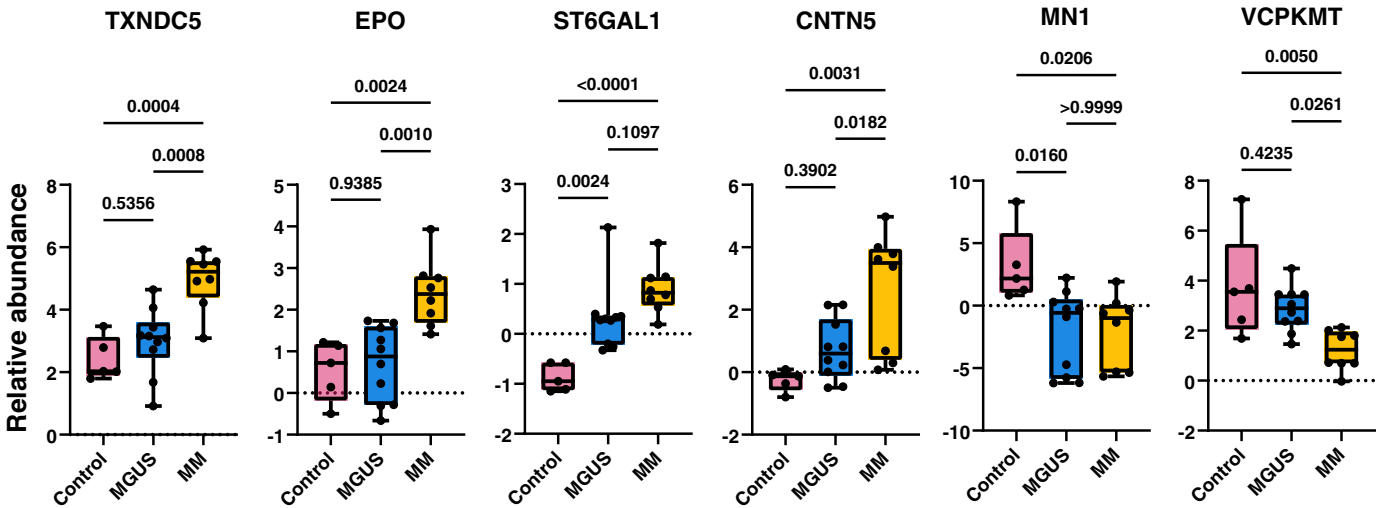

Supplement: Supplementary file 8 — Supplementary Fig. S5 [file 41408_2025_1417_MOESM8_ESM.pdf]
